# Undifferentiated sarcomatoid carcinoma of the pancreas-a single-institution experience with 23 cases

**DOI:** 10.1186/s12885-024-11988-2

**Published:** 2024-02-22

**Authors:** Lei Feng, Xiaojuan Tang, Zhen You

**Affiliations:** Division of Biliary Surgery, Department of General Surgery, West China Hospital, Sichuan University, No.37, Guoxue Lane, Wuhou District, Chengdu, Sichuan China

**Keywords:** Undifferentiated sarcomatoid carcinoma of the pancreas, Surgical, Clinicopathologic features, Overall survival

## Abstract

**Background:**

The clinical course and surgical outcomes of undifferentiated sarcomatoid carcinoma of the pancreas (USCP) remain poorly characterized owing to its rarity. This study aimed to describe the histology, clinicopathologic features, perioperative outcomes, and overall survival (OS) of 23 resected USCP patients.

**Methods:**

We retrospectively described the histology, clinicopathologic features, perioperative outcomes and OS of patients who underwent pancreatectomy with a final diagnosis of USCP in a single institution.

**Results:**

A total of 23 patients were included in this study. Twelve patients were male, the median age at diagnosis was 61.5 ± 13.0 years (range: 35–89). Patients with USCP had no specific symptoms and characteristic imaging findings. The R0 resection was achieved in 21 cases. The En bloc resection and reconstruction of mesenteric–portal axis was undertaken in 9 patients. There were no deaths attributed to perioperative complications in this study. The intraoperative tumor-draining lymph nodes (TDLNs) dissection was undergone in 14 patients. The 1-, 3- and 5-year survival rates were 43.5%, 4.8% and 4.8% in the whole study, the median survival was 9.0 months. Only 1 patient had survived more than 5 years and was still alive at last follow-up. The presence of distant metastasis (*p* = 0.004) and the presence of pathologically confirmed mesenteric–portal axis invasion (*p* = 0.007) was independently associated with poor OS.

**Conclusions:**

USCP was a rare subgroup of pancreatic malignancies with a bleak prognosis. To make a diagnose of USCP by imaging was quite difficult because of the absence of specific manifestations. Accurate diagnosis depended on pathological biopsy, and the IHC profile of USCP was mainly characterized by co-expression of epithelial and mesenchymal markers. A large proportion of patients have an early demise, especially for patients with distant metastasis and pathologically confirmed mesenteric–portal axis invasion. Long-term survival after radical resection of USCPs remains rare.

## Introduction

Pancreatic carcinoma (PC) was an aggressive malignancy that continues to be a leading cause of mortality, with a constantly increasing incidence [1, 2]. Pancreatic ductal adenocarcinoma (PDAC) was the most common type of PC, accounting for more than 90% of cases and showing a very low survival rate [< 10% 5-year overall survival (OS)] [3]. Beyond PDAC, several other morphologically diverse subtypes of PC have been identified. Among these, undifferentiated sarcomatoid carcinoma of the pancreas (USCP) was an extremely rare but very aggressive subtype with a poor prognosis.

According to The World Health Organization (WHO) classification of tumors of the digestive system, USCP represents a subtype of undifferentiated PDAC and accounted for up to 2–3% of all PDAC and its variants [4–6]. Histologically, USCP was predominantly composed of neoplastic spindle-shaped cells with epithelial derivation, showing both epithelial and mesenchymal features [7–9].

Owing to the rarity of the disease, all the available information was originated from individual case reports or small patients’ cohorts. Therefore, the clinicopathologic features, and the therapeutic strategies were poorly characterized. In this study, we aimed to describe the experience at a single institution in the management of USCPs.

## Patients and methods

### Patients and treatment strategy

Retrospective analysis was performed on patients undergoing curable pancreatectomy in West China Hospital of Sichuan University from January 2013 to December 2022. All patients were confirmed to have pancreatic tumors by computed tomography (CT) or magnetic resonance imaging (MRI). A contrast enhanced CT or MRI scan of the abdomen, including arterial, venous, and portal contrast phase axial scans, were required within 2 weeks before surgery. Tumor size, location, and relation to the celiac axis, superior mesenteric artery (SMA) and superior mesenteric vein (SMV), common hepatic artery, and portal vein (PV) were reported. Macrovascular invasion (MaVI) was defined as tumor invasion of the celiac axis, SMA, SMV, PV, and/or common hepatic artery.

Those that meet the following criteria were considered to be resectable or borderline resectable: A tumor without arterial involvement and with venous involvement<90°, or a tumor with arterial involvement<90°and/or venous involvement between 90° and 270° without occlusion. Patients with distant metastases were excluded from the study. However, patients with tumors of the body and tail of the pancreas with splenic metastases were still considered to be eligible for surgery because the spleen needs to be removed at the same time.

### Operative procedure

A total mesopancreatic excision (TMpE) classical or pylorus-preserving pancreatoduodenectomy was performed for pancreatic head tumors. The operative procedure was described in a previously published study by Safi et al. [10]. Reconstruction after pancreaticoduodenectomy depends on the habits of surgeon.

Total pancreatectomy combined with splenectomy (TPS) was performed as previously widely described [11]. The procedure mainly consisted in these following steps. First, mobilization of the whole pancreas through the mobilization of the right colon and hepatic flexure, a wide Kocher maneuver, the gastrocolic ligament division. Second, mobilization of the spleen. In the third step, ligation of the splenic vessels and GDA, then total pancreaticoduodenectomy with cholecystectomy. Finally, in pylorus-preserving cases, choledochojejunostomy and duodenojejunostomy were performed to restore continuity of the gastrointestinal tract. In cases of distal gastrectomy, gastrojejunal anastomosis was performed.

Distal pancreatectomy combined with splenectomy (DPS) was performed for tumors involving the pancreatic body and tail. The procedure of DPS included an en bloc resection of the spleen, the left part of the pancreas and the regional lymph nodes including splenic hilum (station 10), splenic artery (station 11) and inferior border of the pancreatic body (station 18) [12]. An additional LN picking was performed at the coeliac trunk or common hepatic artery in case of suspect adenopathy (enlarged LN, stations 8 and 9) [13].

### Histopathological evaluation

A standardized pathology procedure, on the basis of the Leeds Pathology Protocol, was applied [13], including description of the tumor origin, extension, lymph node metastases, perineural invasion (PNI), and resection margins (RM). RM were considered microscopically positive (R1) if tumor was present at ≤ 1 mm from the transection margins (pancreas, bile duct, stomach, and/or duodenum) or the circumferential dissection (the anterior and posterior sides of the pancreas, the SMA, and the SMV).

Cases were initially identified through a search of our pathology database. The initial diagnosis of the identified patient was consistent with USCP and was reviewed by our pancreatic pathologist. Available cases were independently reviewed and confirmed by two professional pathologists who were blinded to each other, the disagreement was resolved by discussion with a third pathologist. The histopathological diagnosis of USCP was defined as the presence of poorly differentiated or anaplastic cells with a predominance of spindle cells, sarcomatoid features, and epithelial derivation [4]. The tumor staging was performed in accordance with the American Joint Committee on Cancer (AJCC) staging system for PC [14]. Any questionable histopathologic diagnosis underwent pathologic reconfirmation [15].

For patients confirmed with USCP, their demographic characteristics, initial symptoms, clinical manifestations (jaundice, abdominal pain, weight loss, nausea, fever, and vomiting), imaging, and blood chemistry and serological tests, including carbohydrate antigen (CA) 19 − 9 (CA 19 − 9), carcinoembryonic antigen (CEA), cancer antigen 125 (CA-125) were collected retrospectively via chart review.

Perioperative mortality was defined as death within 30 days of the operative date or mortality before hospital discharge. Complications were graded by The Clavien-Dindo Classification of Surgical Complications [16]. OS was calculated from the date of surgery. Date of death was obtained from medical records or telephone interview.

### Statistical analysis

We entered and verified the data using statistical software SPSS (version 26.0). We performed log-rank (Cox-Mantel) survival analyses using Kaplan-Meier methods to test differences in survival (in months) between patients in different groups. Chi-square tests were used to verify the incidence of complications among different patients.

Initially, univariate Cox regression analyses and multivariate proportional hazards regression model were performed to identify independent prognostic factors. Baseline variables that showed a univariate relationship with or that were considered clinically relevant with prognosis were entered into multivariate Cox proportional-hazards regression model. All the variables included were carefully selected, given the small number of cases available, to ensure the simplicity and reliability of the final model. A *p* value of < 0.05 was considered statistically significant for all analyses.

## Results

### Demographic characteristics and clinical presentation

A total of 31 patients had a pathologic diagnosis of USCP and thus were identified in the patient reports, but only 23 of them had complete available information (demographic, clinicopathologic feature, and follow-up) and were thus included in the analysis. Table 1 described the baseline characteristics of this study. Twelve patients were male, the median age at diagnosis was 61.5 ± 13.0 years (range: 35–89). Most patients (60.9%) were aged 60 years or older, more than half of the patients (52.2%) were aged 65 years or older. The majority of patients presented with abdominal pain/discomfort, weight loss, loss of appetite/anorexia and palpable abdominal mass were presented in 4 patients, respectively. The remaining were nausea/vomiting, fatigue, and fever of unknown origin (Table 1).

**Table 1:** Baseline characteristics of the patients with USCP

Variables	No. of patients
Age, median (range), y	61.52 ± 13.01 (35–89)
Sex, male, n (%)	12 (52.2)
Symptoms, n (%)	18 (78.3)
Abdominal pain/discomfort, n (%)	16 (88.9)
Nausea/vomiting, n (%)	2 (11.1)
Weight loss, n (%)	4 (22.2)
Loss of appetite/anorexia, n (%)	4 (22.2)
Fatigue, n (%)	1 (5.6)
Fever of unknown origin, n (%)	2 (11.1)
Palpable abdominal mass, n (%)	4 (22.2)
Preoperative jaundice, n (%)	7 (30.4)
Abnormal of tumor marker, n (%)	
CEA	12 (52.2)
CA19-9	18 (78.3)
CA125	8 (34.8)
Location of tumor, n (%)	
Head	9 (39.1)
Body and tail	14 (60.9)
Tumor size, median (range), mm	62.5 (26–240)
Radiological findings of the tumor, n (%)	
Heterogeneous (cystic tumor combined solid component)	4 (17.4)
Cystic tumor	0
Solid tumor	19 (82.6)
Heterogenous enhancement	18 (78.3)
Tumor density	
Hypodense	23 (100)
Hyperdense	0
Treatment, n (%)	
TMpE Pancreaticoduodenectomy	9 (39.1)
DPS	11 (47.8)
TPS	3 (13.0)

USCP, undifferentiated sarcomatoid carcinoma of the pancreas; CEA, carcinoembryonic antigen; CA19-9, carbohydrate antigen 19 − 9; CA125, cancer antigen 125

### Imaging findings

**Fig. 1:**
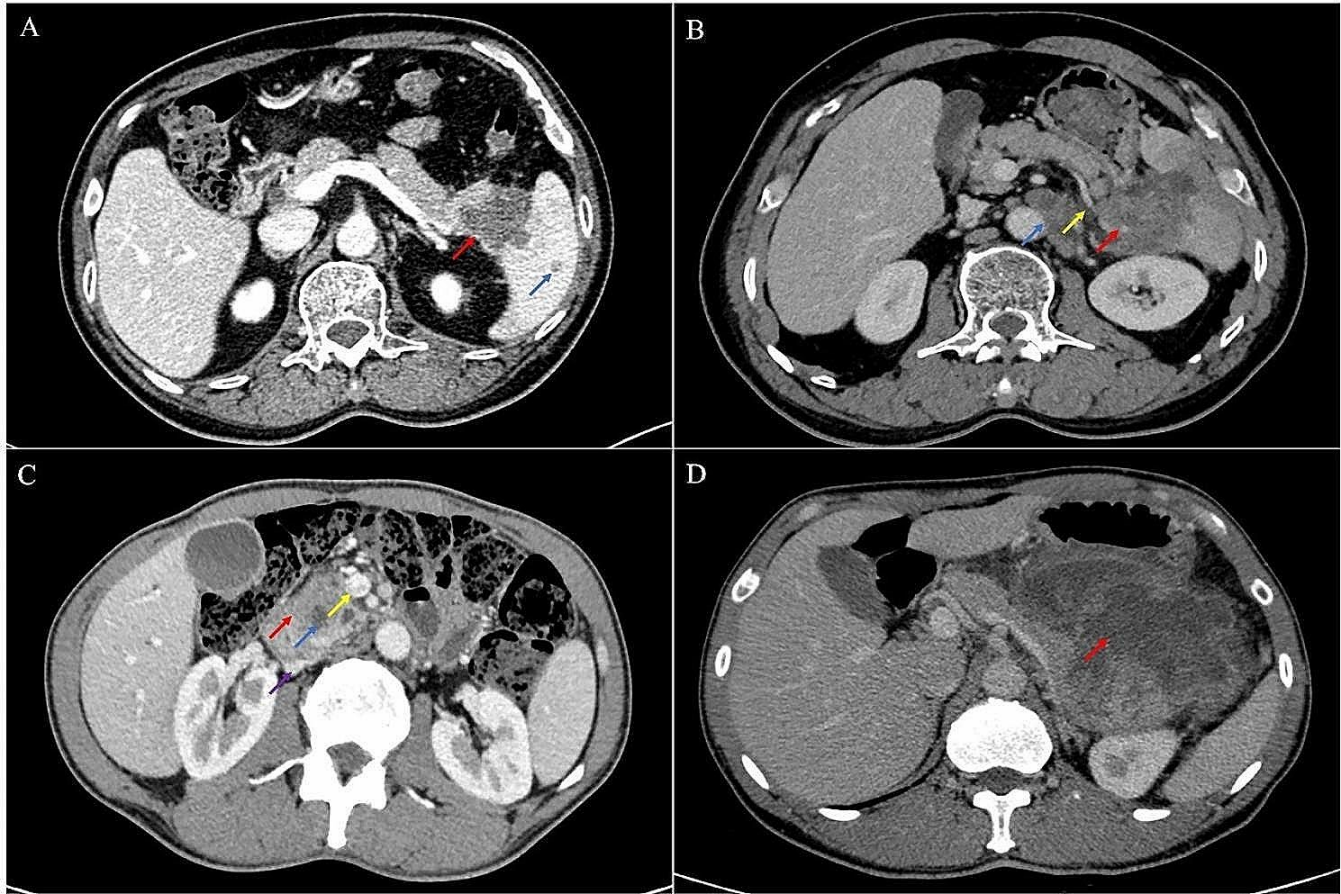
Contrast-enhanced CT scan of the abdomen. **A**. A low density tumor of the pancreatic tail (red arrow) with splenic metastasis (blue arrow). **B**. A tumor located in the tail of the pancreas with ill-defined margins and heterogenous enhancement (red arrow). The splenic artery was encased by the tumor (yellow arrow). Enlarged lymph nodes were seen in the left renal portal and paraaortic (blue arrow). **C**. A slightly low-density tumor with heterogenous enhancement was seen on the head of the pancreas (red arrow), and lower density nodule was seen within the tumor (blue arrow). The tumor compressed the inferior vena cava (purple arrow) and involved the SMV (yellow arrow). **D**. A large tumor originating from the tail of the pancreas. The tumor compresses the tail of the pancreatic body and involves the left kidney. Intratumoral bleeding was observed (red arrow)

Generally, USCP manifested as low-density lesions with ill-defined margins. Three patients presented distant metastasis at diagnosis, all with splenic metastasis (Fig. 1A). Tumor size in greatest dimension was a median of 62.5 mm (mm) with range of 26–240 mm. Most tumors were located in the body or tail of the pancreas (60.9%). A hypodense lesion was appreciated in 23 cases (100%) (Fig. 1B, C), solid appearance in 19 cases (82.6%), heterogenous enhancement in 18 cases (78.3%) (Fig. 1B, C, D). MaVI was found in 13 patients (Fig. 1C). Enlarged tumor-draining lymph nodes (TDLNs) were found in four patients (Fig. 1B). Patient characteristics are represented in Table 1.

In contrast-enhanced CT, the vast majority of patients present with a low-density cystic mass located at the body or tail of the pancreas. Tumor diameter varies greatly among different patients, most patients (82.6%) have tumors larger than 30 mm in diameter, some patients may have intratumoral bleeding (Fig. 1B).

**Fig. 2:**
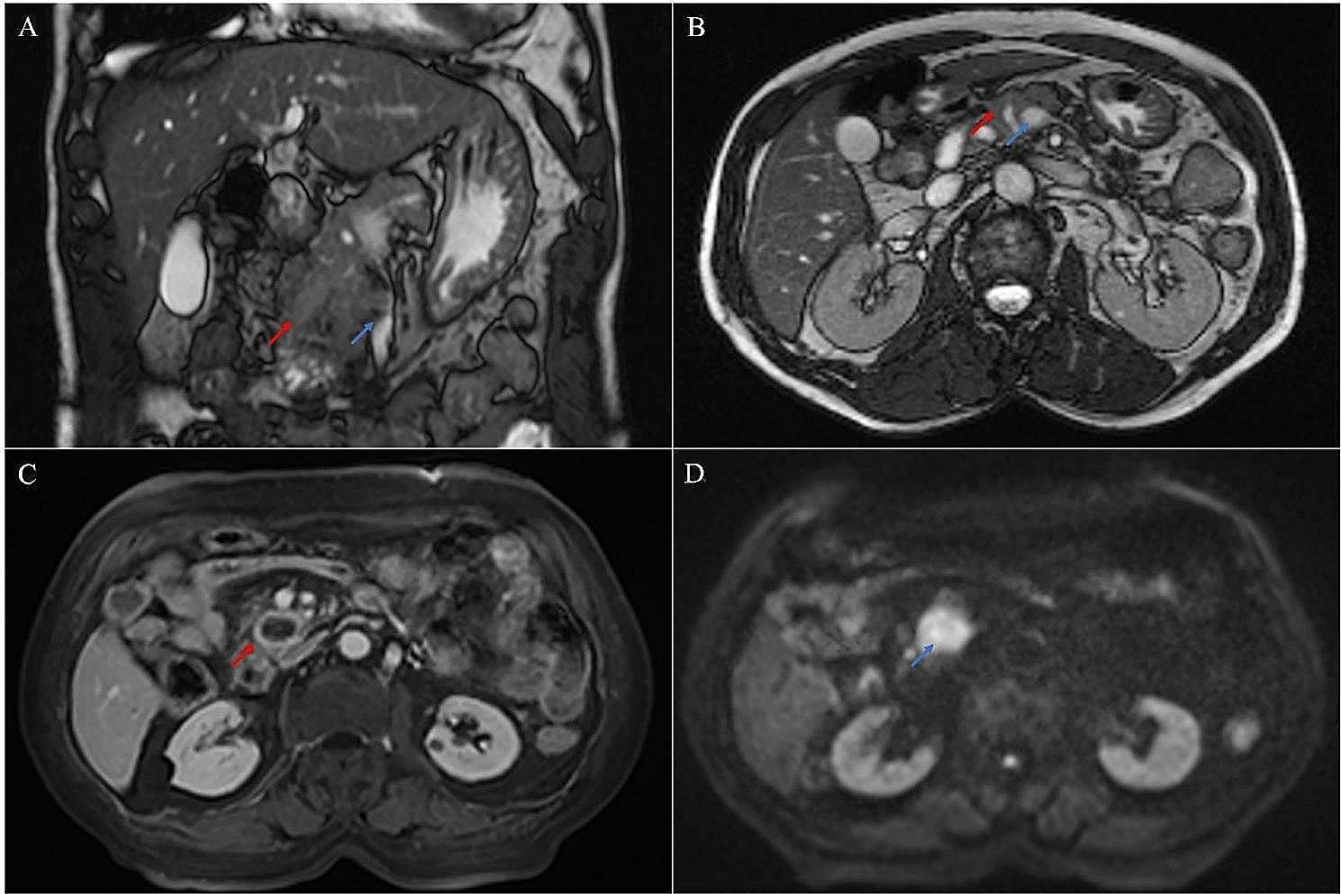
MRI scan of the abdomen. (**A**, **B**). A 71-year-old male patient presented with a low-density tumor located in the uncinate process of the pancreas with ill-defined margins, heterogenous enhancement on enhanced scans (red arrow), and the SMV was encased by the tumor (blue arrow). (**C**, **D**). An 80-year-old male patient presented with a low-density ill-defined margins tumor located on the head of the pancreas, T1WI showed circular enhancement (red arrow), and DWI showed restricted diffusion (blue arrow)

On MRI, USCP mainly presented as low-density lesions with ill-defined margins (Fig. 2A, B), and enhanced scan showed significant circular enhancement on T1-weighted image (T1WI) and T2-weighted image (T2WI) (Fig. 2C). On diffusion-weighted imaging (DWI), the main manifestation was limited diffusion (Fig. 2D).

### Pathological features

**Fig. 3:**
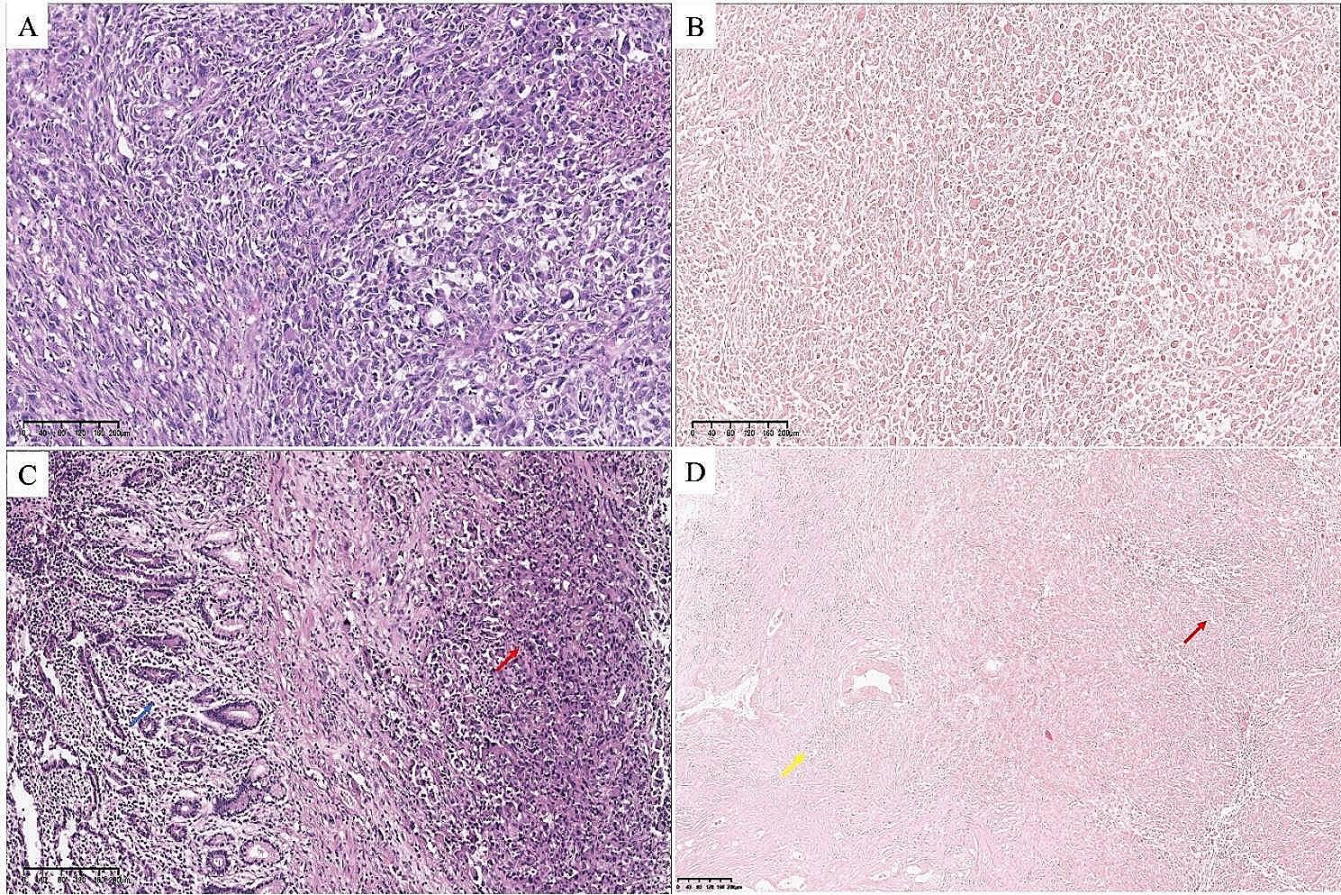
Four paradigmatic images of USCP are presented (H&E staining). (**A**, **B**) Typical hyper-cellular appearance. USCP tends to solid nest-like growth and mainly composed of spindle cells. The tumor cells are poorly differentiated and characterized by the lack of glandular differentiation and the presence of mesenchymal-like, spindle-shaped tumor cells (Original magnification × 100). (**C**, **D**) The H&E staining showed that the tumors were adjacent to each other as two different components: (**C**) sarcomatoid carcinoma (red arrow) and PDAC (blue arrow) and (**D**) sarcomatoid carcinoma (red arrow) and mucinous cystadenocarcinoma of the pancreas (yellow arrow) (Original magnification × 100)

Pathologic analysis revealed a diagnosis of USCP in all cases. Histologically, USCP was a poorly differentiated tumor characterized by the lack of glandular differentiation and the presence of mesenchymal-like, spindle-shaped tumor cells. In hematoxylin eosin (H&E) staining, USCP tended to solid nest-like growth and mainly composed of spindle cells (Fig. 3), often accompanied by perineural invasion (PNI), lymphatic and microvascular infiltration (MVI).

**Fig. 4:**
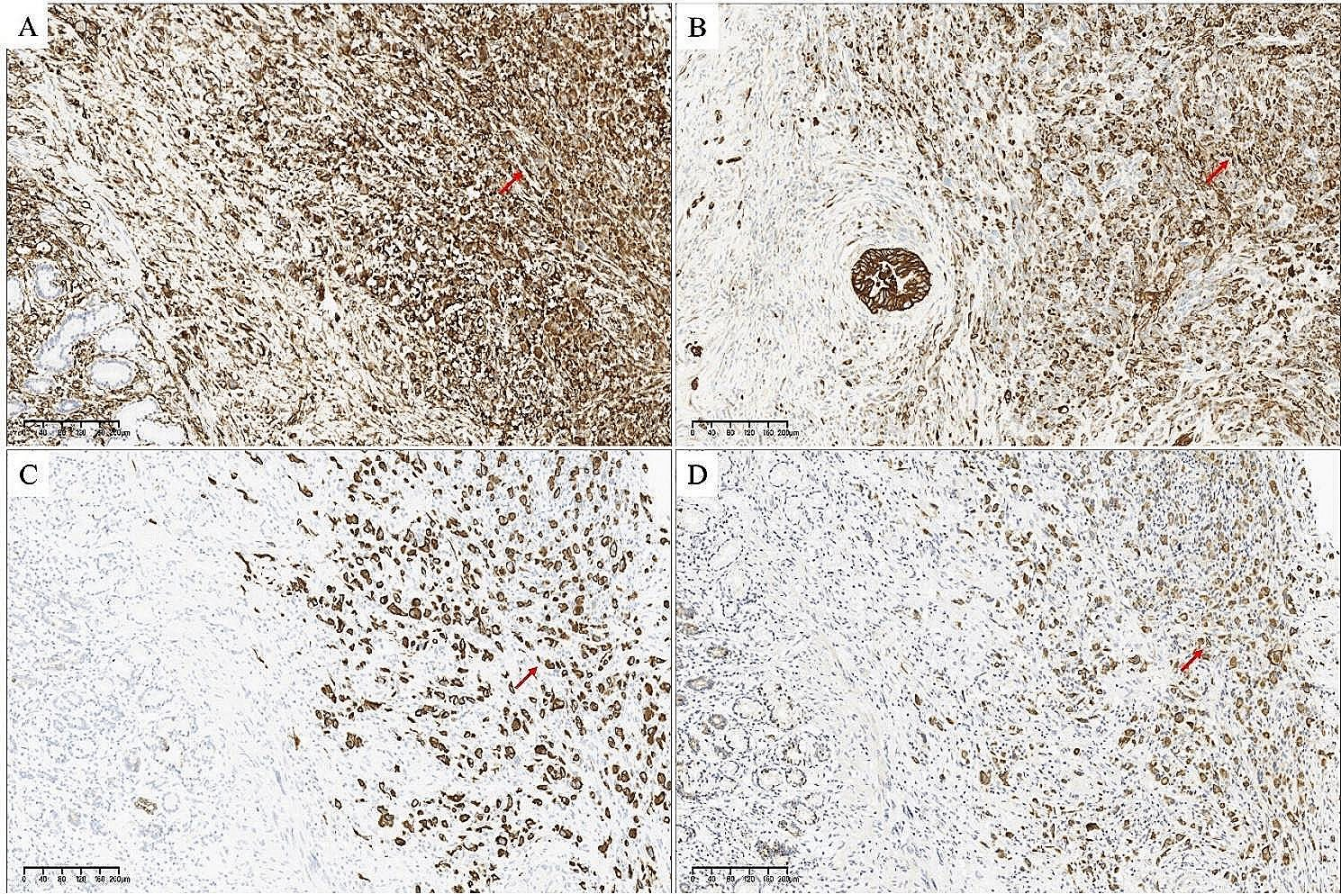
Four paradigmatic images of USCP are presented, the USCP was adjacent to PDAC (IHC staining). (**A**) The USCP components were strongly positive for vimentin (red arrow) (Original magnification × 100). (**B**) The USCP components were strongly positive for PCK (red arrow) (Original magnification × 100). (**C**) The USCP components were focally positive for CK7 (red arrow) (Original magnification × 100). (**D**) The USCP components were focally positive for CK19 (red arrow) (Original magnification × 100)

In IHC staining, all cases were positive for Vimentin (23/23) (Fig. 4A). Pan-cytokeratin (PCK) was found positive in 17 out of 18 patients (17/18) (Fig. 4B), followed by CK7 (16/19) (Fig. 4C), CK19 (14/16) (Fig. 4D), epithelial membrane antigen (EMA) (16/19), CK8 (4/5), CK18 (4/5). Desmin and E-Cadherin (E-Ca) were found positive in only 1 patients (1/11 and 1/9). Eight cases revealed PNI, sixteen patients did not have any lymph node metastasis appreciated, whereas the remaining subjects had at least one lymph node positive for metastatic carcinoma.

### Treatment strategies and follow-up

**Fig. 5:**
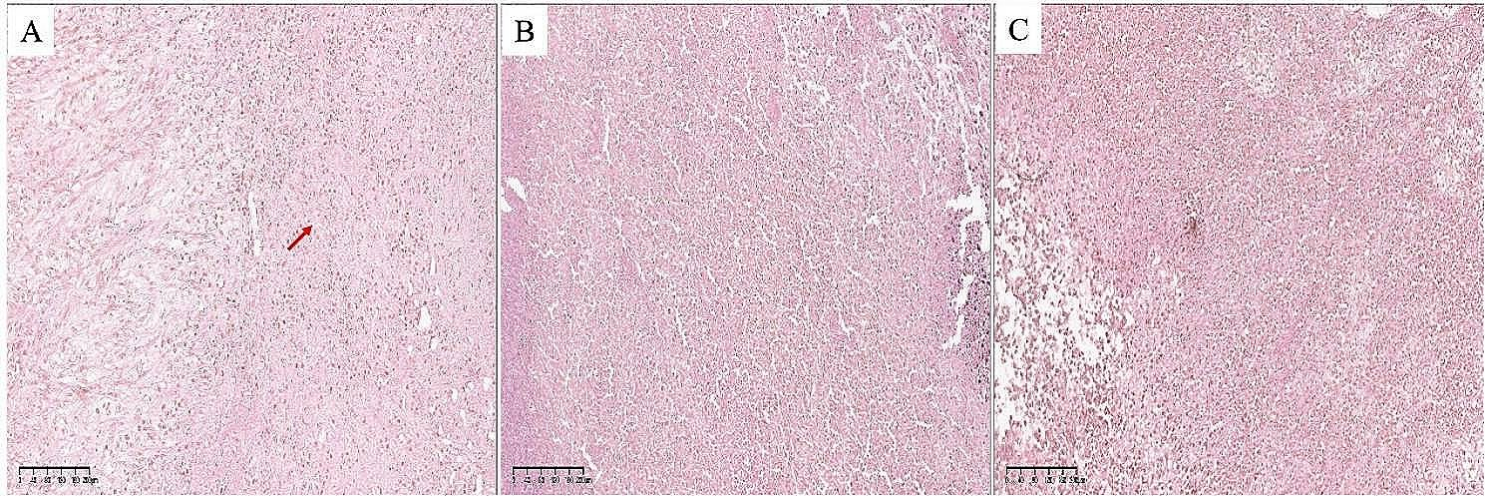
A 69-year-old female USCP patient with liver and gastric metastases. The H&E staining showed poorly differentiated carcinoma, mainly composed by sarcomatoid carcinoma (red arrow) (**A**). The morphology of liver tumor cell (**B**) and gastric tumor cell (**C**) are the same as that of USCP (Original magnification × 100)

Nine patients received pancreaticoduodenectomy (PD), 11 patients received DPS, and 3 patients received TPS The minimally invasive pancreatectomy was performed in 4 patients. The R0 resection was achieved in 21 cases. The En bloc resection and reconstruction of mesenteric–portal axis was undertaken in 9 patients [Inter-national Study Group of Pancreatic Surgery (ISGPS) type 1] [17], but only 2 patients were proved pathologically positive (22.2%). In 5 patients, distant metastases were found during surgery, including liver metastases (Fig. 5B), gastric metastases (Fig. 5C), adrenal metastases, and intestinal metastases. The intraoperative TDLNs dissection was underwent in 14 patients, but only 7 patients were found to have positive lymph nodes. Only 3 patients received PDAC-standardized adjuvant chemotherapy after surgery.

Postoperative complications occurred in 12 patients. Five patients were classified into grade I due to postoperative pain, fever, and electrolyte disturbance. Four patients were classified as grade II due to pulmonary infection, pancreatic fistula, intra-abdominal infection, and total parenteral nutrition (TPN). One patient required ultrasound-guided peritoneal puncture drainage (grade IIIa) due to pancreatic fistula. Two patients had a second operation due to intra-abdominal bleeding (grade IIIb). There were no deaths attributed to perioperative complications in this study.

The difference was not significant in the incidence of complications between patients with or without En bloc resection and reconstruction of mesenteric–portal axis (χ²=2.38, *p* = 0.123), as was between the patients with or without minimally invasive pancreatectomy (χ²=3.05, *p* = 0.081). Also, intraoperative TDLNs dissection did not increase the incidence of postoperative complications (χ²=0.03, *p* = 0.867).

A significant difference was noted between patients with or without the presence of distance metastasis with respect to mean survival [6.29 (95% CI 1.77–10.81) vs. 18.45 (95% CI 10.51–26.39), months, *p* = 0.007], as was between patients with or without pathologically confirmed mesenteric–portal axis invasion [3.00 (95% CI 1.04–4.96) vs. 15.70 (9.28–22.12), months, *p* = 0.016]. There was no significant difference between patients with or without with PDAC-standardized adjuvant chemotherapy respect to mean survival [26.33 (95% CI 21.76–30.91) vs. 12.85 (95% CI 6.10-19.61), months, *p* = 0.092]. No significant difference was noted between patients with or without R0 resection with respect to mean survival [15.05 (95% CI 8.47–21.63) vs. 10.50 (0.00-23.24), months, *p* = 0.559].

### Prognosis

**Fig. 6:**
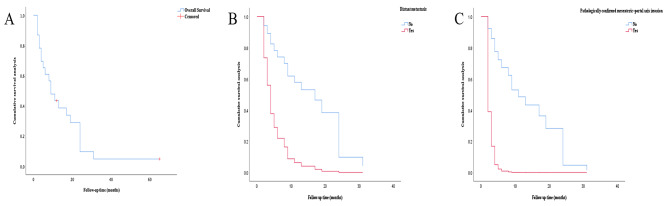
Survival curves of patients. (**A**) The 1-, 3- and 5-year survival rates were 43.5%, 4.8% and 4.8%, the median survival was 9.0 months. (**B**) The presence of distant metastasis was in-dependently associated with poor OS. (**C**) The presence of pathologically confirmed mesenteric–portal axis invasion was in-dependently associated with poor OS

The 1-, 3- and 5-year survival rates were 43.5%, 4.8% and 4.8% in the whole study (Fig. 6A), the median survival was 9.0 months. The duration of follow-up ranged from 2 to 65 months. Two patients were alive at last follow-up and 21 were died of dis-ease (DOD). Only 1 patient had survived more than 5 years and was still alive at last follow-up. The death was attributable to the neoplasm in all of 21 patients and occurred between 2 and 31 months after diagnosis.

When we analyzed the prognostic factors of the tumors, we identified that the presence of distant metastasis [HR = 3.69 (99% CI 1.31–10.37), *p* = 0.013] and the presence of pathologically confirmed mesenteric–portal axis invasion [HR = 5.62 (95% CI 1.08–29.34), *p* = 0.041] as poor predictor of outcome.

Multivariate analysis showed that the presence of distant metastasis [HR = 5.05 (99% CI 1.66–15.37), *p* = 0.004] (Fig. 6B) and the presence of pathologically confirmed mesenteric–portal axis invasion [HR = 11.73 (95% CI 1.95–70.52), *p* = 0.007] (Fig. 6C) were independently associated with poor OS. For favorable prognostic factor, we selected patients without complications, R0-margin, and the receipt of PDAC-standardized adjuvant chemotherapy into multivariate analysis, the result showed that the above factorswas not independently associated with good OS (Table 2).

**Table 2:** Univariate and multivariate analysis for overall survival

Variable	Univariate analysis		Multivariate analysis	
	HR (95% CI)	*p* value	HR (95% CI)	*p* value
Gender	1.69 (0.71–4.04)	0.240	Not selected	Not selected
Age group				
≥60	1.65 (0.64–4.25)	0.296	Not selected	Not selected
≥65	0.68 (0.28–1.66)	0.396	Not selected	Not selected
Location	1.28 (0.52–3.11)	0.592	Not selected	Not selected
PNI	1.38 (0.54–3.48)	0.502	Not selected	Not selected
MaVI	0.88 (0.36–2.14)	0.782	Not selected	Not selected
T-category				
T1	N/A	N/A	N/A	N/A
T2	1.09 (0.43–2.76)	0.855	Not selected	Not selected
T3	0.66 (0.26–1.64)	0.655	Not selected	Not selected
T4	5.62 (1.08–29.34)	0.041	15.22 (2.27-101.82)	0.005
N-category				
N1	0.75 (0.27–2.07)	0.575	Not selected	Not selected
N2	11.00 (1.00-121.31)	0.050	3.94 (0.19–83.40)	0.379
M-category	3.69 (1.31–10.37)	0.013	4.24 (1.28–14.05)	0.018
Resection Margin Status				
R0	1.54 (0.35–6.85)	0.571	1.27 (0.28–5.86)	0.758
R1	1.54 (0.35–6.85)	0.571	Not selected	Not selected
Lymph node metastasis	0.55 (0.22–1.39)	0.204	Not selected	Not selected
Pathologically confirmed mesenteric–portal axis invasion	5.62 (1.08–29.34)	0.041	15.22 (2.27-101.82)	0.005
Disease stage				
IA	N/A	N/A	N/A	N/A
IB	1.35 (0.46–3.96)	0.587	Not selected	Not selected
IIA	1.89 (0.06–0.62)	0.006	7.30 (1.76–30.29)	0.006
IIB	0.77 (0.18–3.32)	0.722	Not selected	Not selected
III	5.62 (1.08–29.34)	0.041	Not selected	Not selected
IV	3.69 (1.31–10.37)	0.013	3.15 (1.02–9.75)	0.046
Laparoscopic approach	1.33 (0.44–4.06)	0.613	Not selected	Not selected
Reconstruction of mesenteric–portal axis	1.42 (0.60–3.40)	0.428	Not selected	Not selected
Complications	1.02 (0.42–2.46)	0.963	Not selected	Not selected
I	1.71 (0.57–5.14)	0.337	Not selected	Not selected
II	1.08 (0.36–3.26)	0.895	Not selected	Not selected
IIIa	0.37 (0.05–3.03)	0.355	Not selected	Not selected
IIIb	5.62 (1.08–29.34)	0.041	2.25(0.22–23.47)	0.498
Elevated preoperative CA19-9	1.30 (0.43–0.64)	0.647	Not selected	Not selected
Preoperative CEA	1.78 (0.70–4.55)	0.227	Not selected	Not selected
Preoperative CA125	1.54 (0.61–3.89)	0.363	Not selected	Not selected
PDAC-standardized adjuvant chemotherapy	0.39 (0.11–1.36)	0.139	0.28 (0.06–1.23)	0.092

HR, hazard ratio; CI, confidence interval; CEA, carcinoembryonic antigen; CA19-9, carbohydrate antigen 19 − 9; CA125, cancer antigen 125; PDAC, pancreatic ductal adenocarcinoma

## Discussion

USCP was a rarely observed malignant subtype of undifferentiated carcinoma characterized by a predominance of spindle cells and sarcomatous morphologic features with epithelial derivation [4, 18–20]. USCP most commonly in patients over 60 years of age, with an average age of about 61.7 years at diagnosis, the incidence is almost equal between men and women [21]. In this study, the clinical presentations of patients were not specific, and about 21.7% of patients were found by chance.

Contrast-enhanced CT examination is the best non-invasive imaging method for pancreas examination at present with good spatial and temporal resolution, mainly used for the diagnosis, differential diagnosis and staging of USCP. Contrast-enhanced CT can show the size, location, morphology, internal structure and the relationship between pancreatic tumors and surrounding structures and can accurately determine whether there is liver metastasis and show enlarged TDLNs.

With the improvement of MR Scanning technology and the improvement of temporal and spatial resolution, the quality and diagnostic accuracy of MRI and magnetic resonance cholangiopancreatography (MRCP) have been greatly improved, showing increasing value in displaying USCP, judging vascular invasion, accurate clinical staging, and other aspects. At the same time, MRI has the characteristics of multi-parameter, multi-plane imaging without radiation, and can be used as a useful supplement to CT enhanced scanning when the differential diagnosis of pancreatic lesions is difficult. When patients are allergic to CT enhanced contrast agents, MRI can be used instead of CT scan for diagnosis and clinical staging. The application of MRCP and multi-phase enhanced scanning was more advantageous in the qualitative and differential diagnosis of USCP. MRCP can clearly show the full picture of the pancreatic cholangiopancreatography system, help to judge the lesion site, and thus contribute to the detection and differential diagnosis of periampullary carcinoma. Compared with endoscopic retrograde cholangiopancreatography (ERCP), MRCP has non-invasive advantages.

Contrast-enhanced CT and MRI can be used to assess the stage of USCP and the involvement of vascular. The degree of contact between the tumor and local vascular was classified as uninvolved, abutted, or encased [22].

The abutment indicated that vascular involvement was not exceeding 180° (Fig. 1C), and the encasement indicated that vascular involvement was greater than 180° (Fig. 2A, B). This can provide vital information to define the most optimal initial treatment.

Common sites of PDAC metastases were liver (90%), lymph nodes (25%), lung (25%), peritoneum (20%), and bones (10–15%) [23]. Metastases were confirmed in at least 14 patients (including distant metastasis and TDLNs metastasis), involving a total of 17 sites. The most common metastases of USCP in this study was TDLNs (41.2%), spleen (17.6%), liver (11.8%), stomach (11.8%), left adrenal gland (LAG) (11.8%) and small intestine (5.9%).

Alguacil-Garcia et al. first divided anaplastic PC into four subsets, including round cell anaplastic, spindle cell, pleomorphic giant cell, and osteoclastic giant cell [19]. However, many cases exhibited a range of histopathological features, so classifying an individual case into one of these categories remains quite challenging.

USCP can exhibit an appearance of monophasic or biphasic. The monophasic pattern was often referred to as spindle cell carcinoma, similar to a soft tissue sarcoma without epithelioid areas. The biphasic pattern is characterized by a mixture of mesenchymal and epithelioid cells with a transitional zone (Fig. 3C, D). The sarcomatous tissue of these tumors also showed a tendency toward epithelial-oriented differentiation rather than specific mesenchymal differentiation [24].

Accurate pathological diagnosis was quite difficult, and IHC was still the primary diagnostic method [25, 26]. In IHC, undifferentiated cells typically express broad lineage carcinoma (PCK) and sarcoma (vimentin and desmin) markers and exhibit the absence of E-Ca [27].

The occurrence of sarcomatoid carcinoma may be related to the epithelial-mesenchymal transition (EMT). EMT was not a unidirectional switch between two distinct cell states, but a transitional state between the extreme epithelial and mesenchymal endpoints. As a result, the tumor cells end up behaving like mesenchymal cells but retain some of the key epithelial markers [28]. The activation of EMT was a key process in the metastasis of cancer cells, during which epithelial cells acquire mesenchymal cell characteristics and enhanced cell motility and migration.

E-Ca was an indicator of the EMT during the metastatic of carcinoma, decreased expression of E-Ca expression was the fundamental event of EMT and tumor metastasis [29], moreover, loss of E-Ca expression promoted cancer cell metastasis through multiple downstream transcriptional pathways [30].

Vimentin was a type III intermediate-filament protein that, together with microtubules and microfilaments, formed the skeletal structure of cells. Vimentin staining can be used as a marker for cells of interstitial origin or for cells developing EMT. Due to its many roles in the cell, vimentin can be studied in many disciplines, from cancer to cytoskeletal dynamics. Cancer cells often exhibited EMT and other characteristics during metastasis, and vimentin contributes to EMT by changing cell shape and movement. The expression of vimentin was up regulated during EMT, and the overexpression of vimentin was associated with increased aggressiveness and metastasis in a variety of cancers [31]. Desmin was used to identify tumors with myoid differentiation and was mostly negative in epithelioid malignant mesothelioma.

PNI, a process by which tumors invade peripheral nerves, was a common mode of metastasis for tumors, found in approximately 80–100% of patients with PC, and was closely associated with poor prognosis [32]. PNI was found positive in only 34.8% patients in this study, no significant difference was noted between patients with or without PNI with respect to mean survival [15.75 (95% CI 10.58–20.92) vs. 13.53 (5.16–21.91), months, *p* = 0.467]. Moreover, when we analyzed the prognostic factors of the tumors, we found that PNI was not independently associated with poor OS.

The treatment of USCP remains challenging, even after radical resection, the median survival time rarely exceeds one year [18, 33–36]. The optimal surgical approach depended on the location of the tumor and its relationship to the bile duct and blood vessels. The surgical procedure did not affect the prognosis of the patient with USCP, and minimally invasive pancreatectomy has been demonstrated to be safe with a complication rate similar to that of open pancreatectomy.

Patients with MaVI invasion may require vascular resection and/or reconstruction. A negative incisal margin should be the primary requirement for pancreatectomy. The En bloc resection and reconstruction should be performed in patients with tumor invasion into the mesenteric–portal axis to obtain a R0 resection, this was a standard therapy for patients with borderline resectable PDAC (BRPC) [37], and the prognosis was similar in patients with or without tumor invasion of the mesentery-portal axis. Although En bloc resection and reconstruction of mesenteric–portal axis did not lead to an increased incidence of complications, only a small number of patients were pathologically confirmed to have tumor invasion. The presence of pathologically confirmed mesenteric–portal axis invasion was an independently poor survival prognostic factor. However, it was quite difficult to evaluate which tumors have true vascular invasion by preoperative imaging, even during surgery procedure, most patients actually take unnecessary risks for this.

The TDLNs were critical sites to elicit anti-tumor immunity. However, due to the direct inflow of lymph from the drainage area, TDLNs can also be colonized by metastatic tumor cells and cause immunosuppression. TDLNs metastases can also serve as a source of hematogenous metastasis. Therefore, intraoperative dissection of TDLNs can potentially reduce the burden of systemic metastasis. However, the dissection of TDLNs impairs the systemic anti-tumor immune response, making patients prone to lymphedema and increasing the probability of postoperative complications. Intraoperative lymph node dissection did not increase the incidence of postoperative complications.

The metastasis was quite common in USCP patients. Unlike PDAC, the most common metastatic site of USCP was the TDLNs. Among the distant metastases, splenic metastases occupy the first place (30%). Reiter et al. found that distant metastases are typically monophyletic and genetically similar to each other (Fig. 5A, B, C). TDLNs metastases, in contrast, display high levels of inter-lesion diversity [38].

There were often conflicting results in different reports on the effect of marginal status on the prognosis of PC patients [39]. Our study confirmed that achieving R0 resection or not did not affect the mean survival of patients. Moreover, an R0 resection was not associated with a good outcome. The limited number of patients in this study may assessed hamper comparability.

In our study, none of the patients died from perioperative complications. Symptoms in most patients were mild, such as fever, pain, or electrolyte disturbance, which can be corrected with medication. The occurrence of perioperative complications did not affect the OS of patients.

Long-term survival after resection for PDAC approached 10% [22]. The presence of the following prognostic factors predicted poorer prognosis: advanced T-category, the presence of TDLNs metastasis or distant metastasis, the presence of gross or microscopic residual disease, high histologic grade, invasion of MaVI, and poor performance status (PS) [40]. Due to the rarity of USCPs, previous studies lacked the statistical power to make a comparative analysis of these factors. In our study, the largest volume to date, we reported 23 patients with USCPs at one time. We found that the presence of distance metastasis and the presence of pathologically confirmed mesenteric–portal axis invasion predicting poorer survival. The only patient to achieve long-term survival in our series underwent distal pancreatectomy with R0 resections and received radical lymph node dissection (15 lymph nodes).

USCP were still rarely described and poorly understood so far, most existing literatures convey a OS worse than that of PDAC [21]. Our study confirmed the notion that a large proportion of patients have an early demise, and long-term survival after radical resection of USCP remains rare. However, a diagnosis of USCP did not necessarily portend a bleak prognosis, and patients still have a chance of a good prognosis with proper treatment.

## Conclusion

USCP was a rare subgroup of pancreatic malignancies with a bleak prognosis. To make a diagnose of USCP by imaging was quite difficult because of the absence of specific manifestations. Accurate diagnosis depended on pathological biopsy, and the IHC profile of USCP was mainly characterized by co-expression of epithelial and mesenchymal markers. A large proportion of patients have an early demise, especially for patients with distant metastasis and pathologically confirmed mesenteric–portal axis invasion. Long-term survival after radical resection of USCPs remains rare.

## Data Availability

The data underlying this article will be shared on reasonable request to the corresponding author.
